# Patients with Duchenne and Becker muscular dystrophies are not more asymmetrical than healthy controls on timed performance of upper limb tasks

**DOI:** 10.1590/1414-431X20176031

**Published:** 2017-07-24

**Authors:** M.C. Artilheiro, C.S.C. Sá, F.M. Fávero, F.A. Caromano, M.C. Voos

**Affiliations:** 1 Departamento de Fonoaudiologia, Fisioterapia e Terapia Ocupacional, Faculdade de Medicina, Universidade de São Paulo, São Paulo, SP, Brasil; 2 Departamento de Ciências do Movimento Humano, Universidade Federal de São Paulo, Santos, SP, Brasil; 3 Departamento de Neurologia/Neurocirurgia, Departamento de Neurologia Clínica, Universidade Federal de São Paulo, São Paulo, SP, Brasil

**Keywords:** Duchenne muscular dystrophy, Upper extremity, Evaluation, Physical therapy

## Abstract

This study aimed to investigate possible asymmetries and relationships between performance of dominant and non-dominant upper limbs (UL) in patients with Duchenne and Becker muscular dystrophies (DMD/BMD), to compare UL performance of patients and healthy subjects and to investigate the relationship between timed performance of UL and age, motor function and muscle strength in DMD/BMD patients. Sixteen patients with DMD and 3 with BMD were evaluated with Jebsen-Taylor Test (timed performance), Vignos scale and Dimension 3 of Motor Function Measure (motor function), and Medical Research Council scale (muscle strength) on a single session. ANOVA showed no asymmetry between dominant and non-dominant UL, except in the writing subtest, in patients and in healthy controls. There were relationships between dominant and non-dominant UL performances. Correlations between timed performance, motor function and muscle strength were found, but age was not correlated with these variables. These findings may reduce the assessment time, prevent fatigue and provide more accurate clinical reasoning involving UL in DMD/BMD treatment.

## Introduction

The recent increase of life expectancy of patients with Duchenne and Becker muscular dystrophies (DMD and BMD) represents more patients living with impaired upper limb (UL) function for longer times (1). DMD and BMD are related conditions that affect skeletal muscles. They occur almost exclusively in males and have similar signs and symptoms that are caused by different mutations in the same gene. Because DMD and BMD result from faulty or missing dystrophin, these conditions are classified as dystrophinopathies.

DMD and BMD may differ in severity, age of onset and rate of progression. In DMD, muscle weakness tends to decrease faster. DMD and BMD together affect 1 in 3500 to 5000 newborn males worldwide. Both show proximal-to-distal progression, with relative sparing of distal hand function (2). Losses of hand and wrist functions occur mainly in advanced stages (3,4). Due to the progressive nature of DMD/BMD, upper extremity weakness and limitations impair the ability of performing daily life activities, including eating, bathing, and tooth brushing (2,5).

Assessing UL function in non-ambulant patients with DMD/BMD is challenging, because of the combination of severe weakness, contractures, clinical heterogeneity and varied compensatory strategies (from patient to patient and even from limb to limb) (1). Janssen et al. (6) investigated the differences between dominant and non-dominant UL activity of shoulder and elbow muscles of 5 patients with DMD. The patients performed single movements of these joints and the non-standardized tasks of reaching forward, touching the contralateral shoulder, combing hair and bringing the hand to the mouth. They found no significant differences in shoulder and elbow muscle recruitment between ULs (6). However, the activities of daily living were just simulated, without real goal or use of objects, which may have interfered on the results. Although non-dominant UL performance seems to be poorer than dominant limb performance, no studies compared the performance of patients with DMD/BMD in real and goal-directed functional activities (7).

Considering that scoliosis is frequent in DMD/BMD, the convexity could be related to UL asymmetry (3). Additionally, a better understanding about UL performance and possible symmetries or asymmetries between dominant and non-dominant limbs could optimize evaluation and clinical reasoning for interventions involving UL function in DMD/BMD (7).

This study aimed to investigate possible differences and relationships between the performances of dominant and non-dominant ULs in daily living, goal-directed activities in patients with DMD/BMD. We hypothesized that dominant and non-dominant ULs would exhibit significantly different performances. Also, we aimed to compare patients with normative data from the literature of gender- and age-matched healthy controls. We hypothesized that UL asymmetry would be higher in patients with DMD/BMD than in healthy controls, because patients show many compensatory movements.

As a secondary objective, we aimed to investigate the relationship of UL timed performance and age, motor function and muscle strength in DMD/BMD patients. We hypothesized that these variables would be related in DMD/BMD patients and that the asymmetry would be less evident in initial DMD/BMD (younger patients) than in severe cases (older patients).

## Material and Methods

The study was approved by the Federal University of São Paulo Ethics Committee (process No. 132-193). This is a prospective observational study with cross-sectional design.

### Subjects

All patients from the Brazilian Association of Muscular Dystrophy (ABDIM) with non-normal levels of dystrophin detected by DNA analysis were recruited to participate. The inclusion criterion was the confirmation of absence (in DMD) or decreased levels (in BMD) of dystrophin, which is a biomarker of a genetic error on Xp21 (8). Forty-three patients gave informed consent to participate.

Ten patients were excluded due to time unavailability. Seven patients were excluded because they could not grasp objects. Seven patients were excluded due to difficulty understanding the tasks, caused by severe cognitive impairment, detected by the Mini-Mental State Examination (MMSE) (9). In a previous pilot study, we observed that a cut-off of ten points (instead of eighteen) could be considered for these patients, because they could perform all Jebsen-Taylor and Motor Function Measure tasks. All patients included in the study were able to understand the tasks (10).

The patients, parents or legal guardians signed the informed consent form prior to participation. Sixteen patients with DMD (aged 16.8±3.9 years) and 3 with BMD (aged 26.0±1.7 years) were evaluated. All patients scored 7 or 8 on the Vignos Scale (11) and used wrist orthosis during the night.

### Procedures

Lower extremity function was classified by the Vignos Scale (11). The scores range from 1 to 10, with 1 meaning that the subject can walk and climb stairs without assistance, and 10 meaning that the subject is confined to a bed.

Upper extremity function was classified by the Brooke Scale (12). This scale ranges from 0 to 6, with 1 meaning that the patient is able to abduct the shoulder in 180 degrees and 6 meaning that the patient has no useful function of the UL.

Dominance was determined by asking and observing the preferred hand during the manipulation of everyday objects, such as scissors, comb, toothbrush and pencil (13).

Muscle strength of shoulders (flexors, extensors, abductors, adductors, internal and external rotators), elbows (flexors, extensors, supinators and pronators) and wrists (flexors and extensors) were tested by the Medical Research Council (MRC) Scale (14). To test muscle groups, manual resistance was applied with the patient lying on dorsal, ventral, and lateral decubitus, and on sitting position. The score was graded from 0 to 5, with 0 indicating no muscle contraction and 5 indicating normal strength (15). We adopted the MRC index (14) to calculate the percentage of muscle strength according to proximal (shoulder) or distal (elbow and wrist) location.

Spinal deformity was evaluated by asking the patient to lean forward with support if necessary (16). The side (right or left) and curve convexity were registered.

All patients were assessed with the Jebsen-Taylor Test (JTT) (17,18) and Dimension 3 of Motor Function Measure (MFM D3) (19–21). Each patient was positioned on his own wheelchair or in a height-adjustable chair, which allowed the correct positioning of hips, knees and ankles, flexed at approximately 90°. A table with adjustable height was used. Each subtest was conducted after making sure the patient had understood the task.

JTT has seven subtests (1: writing; 2: turning over cards; 3: picking up small common objects; 4: simulating feeding; 5: stacking checkers; 6: picking up large light objects; 7: picking up large heavy objects). All subtests have gender- and age-matched normative values for people between 15 and 25 years old in order to compare timed performances (22). Subtest 7 was excluded because patients with DMD/BMD have difficulty carrying heavy objects. Subtests 1, 2, 3, 4, 5, and 6 were performed in sequence, first with the non-dominant UL and then with the dominant UL, according to Jebsen-Taylor standard instructions (17). The time of all attempts was measured in seconds (s) using a stopwatch (17,18). When a patient was not able to complete a JTT subtest due to motor difficulties, the score was registered as the highest time on the subtest plus 20%.

MFM D3 consists of seven items to evaluate distal motor function. The scoring of each item uses a 4-point scale (from 0 to 3). Zero means that the patient cannot initiate the task or cannot maintain the starting position and 3 means the patient is able to complete the task with the standard pattern. The total score is reported as a percentage of the maximum possible score (19–21).

### Data analysis

Data showed homoscedasticity (tested by Levene Test) and normal distribution (tested by Kolmogorov-Smirnov Test). Analyses of variance (two-way ANOVAs) compared dominant and non-dominant UL timed performances. When *post hoc* analyses were necessary, Tukey tests were run. The relationships between dominant and non-dominant UL performances and JTT, MFM D3 and MRC scores were tested by Pearson correlation tests. The correlation coefficients (r) were considered strong when r≥0.70 and moderate when 0.70>r≥0.40 (23).

In order to investigate UL asymmetry in JTT subtests, patients were compared to normative data. A dominance index (the mean time of the dominant UL divided by the mean time of the non-dominant UL) was calculated, which represents UL symmetry. Values near zero indicate UL asymmetry and values near one indicate UL symmetry. The comparison between two proportions was done with the chi-square test. The relationships between the dominance indexes and age were tested by Spearman correlation tests (23).

Statistical analysis was performed using the Statistica software version 13.0 (Statsoft, USA). The level of significance was set at 5%.

## Results

### Clinical characteristics

Sample characteristics are displayed in Table 1. Vignos scores ranged from 1 to 8 and Brooke scores ranged from 1 to 5. About 90% (n=17) of patients exhibited right UL dominance. The median of the percentages of proximal muscle strength and distal muscle strength were 50 and 66%, respectively. Most patients (n=10; 52.6%) showed thoracolumbar scoliosis, 5 patients (26.3%) showed lumbar scoliosis, 1 (5.3%) showed thoracic scoliosis and 3 (15.8%) did not show scoliosis. All patients understood and performed all tests without referring pain, fatigue or discomfort.

**Table 1.** Characteristics of study sample.

Patient	Age (years)	MMSE (score)	Vignos (score)	Brooke (score)	Proximal/Distal muscle strength	Diagnosis	Dominance	Scoliosis description
1	13	28	1	1	80/97%	DMD	R	Absent
2	16	28	8	3	47/62%	DMD	R	Thoracolumbar R
3	20	28	8	5	27/57%	DMD	R	Thoracolumbar R
4	16	28	8	3	43/53%	DMD	R	Thoracolumbar L
5	28	28	2	1	87/87%	BMD	R	Absent
6	17	26	7	3	50/80%	DMD	L	Thoracolumbar R
7	18	27	8	3	50/63%	DMD	L	Thoracolumbar L
8	25	21	8	3	47/70%	BMD	R	Lumbar L
9	24	16	7	3	47/63%	DMD	R	Thoracolumbar L
10	11	13	1	1	83/82%	DMD	R	Absent
11	20	15	8	5	33/50%	DMD	R	Lumbar L
12	13	30	2	1	70/80%	DMD	R	Thoracolumbar R
13	25	28	6	3	60/62%	BMD	R	Thoracolumbar L
14	13	24	3	2	63/97%	DMD	R	Thoracic R
15	15	30	7	3	50/65%	DMD	R	Lumbar R
16	16	12	8	3	60/60%	DMD	R	Thoracolumbar R
17	23	27	7	4	30/57%	DMD	R	Lumbar L
18	21	29	7	3	50/85%	DMD	R	Lumbar R
19	13	11	7	2	50/65%	DMD	R	Thoracolumbar R
Descriptive data	mean±SD 18.3±5.0	mean±SD 23.6±6.7	median7	median3	median50/66%	16 DMD/3 BMD	17 R/2 L	10 Thoracolumbar/5 lumbar/3 absent/1 thoracic
Minimum-maximum	11–28	11–30	1–8	1–5	27–87%/50–97%	–	–	–

DMD/BMD: Duchenne and Becker muscular dystrophies; MMSE: Mini-Mental State Examination; R: right; L: left.

### Comparison between dominant and non-dominant UL performance on JTT subtests

**Figure 1.**
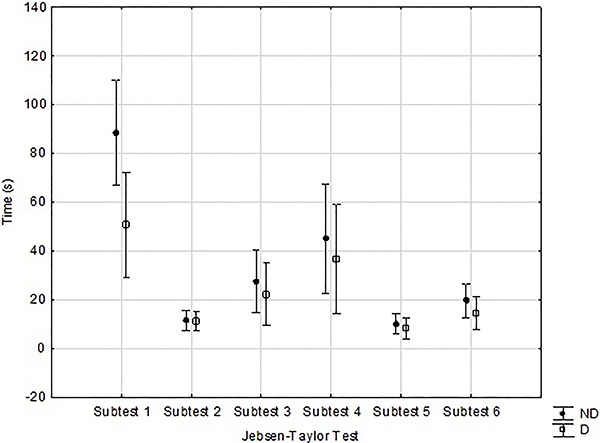
Repeated measures analysis of variance reported as means and 95% confidence intervals for each subtest of Jebsen-Taylor Test, comparing non-dominant (ND) and dominant (D) upper limbs. There was an interaction between subtest and limb (P<0.001) and *post hoc* Tukey tests showed a significant difference between ND and D upper limbs (P<0.001) only in subtest 1.

Repeated measures ANOVA showed an effect of JTT subtest (F_5,90_=17.167; P<0.001; ES=0.488), an effect of UL (F_1,18_=15.444; P<0.001; ES=0.462) and an interaction between subtest and UL (F_5,90_=10.433; P<0.001; ES=0.367). *Post hoc* Tukey tests showed a significant difference between non-dominant and dominant UL only in subtest 1 (P<0.001; Figure 1).

No significant differences between patients and controls (normative data) were found in the dominance indexes of JTT subtests (Table 2).

**Table 2.** Comparison between dominance index of healthy subjects and Duchenne and Becker muscular dystrophies (DMD/BMD) patients, based on Jebsen-Taylor subtests.

Subtest/Dominance	Healthy subjects		DMD/BMD patients		P
	Mean time	Dominance index	Mean time	Dominance index	
1					
NDUL	20.06	0.414	88.47	0.541	0.193
DUL	8.31		47.89		
2					
NDUL	6.47	0.912	11.33	0.986	0.142
DUL	5.9		11.17		
3					
NDUL	6.85	0.873	27.48	0.832	0.345
DUL	5.98		22.86		
4					
NDUL	7.29	0.809	45.03	0.815	0.479
DUL	5.9		36.69		
5					
NDUL	3.75	0.885	9.99	0.818	0.256
DUL	3.32		8.17		
6					
NDUL	4.14	0.855	19.56	0.739	0.155
DUL	3.54		14.45		

NDUL: non-dominant upper limb; DUL: dominant upper limb. P*>*0.05, ANOVA.

### Correlations between dominant and non-dominant UL performances on JTT

Strong positive correlations were found between non-dominant and dominant UL in all JTT subtests (Table 3). Many strong relationships were found between the performances of dominant and non-dominant UL in subtests 2, 3, 4, 5, and 6 (Table 3).

**Table 3.** Correlations between times of subtests of Jebsen-Taylor Test performed by dominant and non-dominant upper limbs.

	1 ND	1 D	2 ND	2 D	3 ND	3 D	4 ND	4 D	5 ND	5 D	6 ND	6 D
1 ND	–	–	–	–	–	–	–	–	–	–	–	–
1 D	**0.948**	–	–	–	–	–	–	–	–	–	–	–
2 ND	0.371	–	–	–	–	–	–	–	–	–	–	–
2 D	–	0.228	**0.869**	–	–	–	–	–	–	–	–	–
3 ND	0.085	–	**0.889**	–	–	–	–	–	–	–	–	–
3 D	–	0.394	–	**0.713**	**0.713**	–	–	–	–	–	–	–
4 ND	0.141	–	**0.760**	–	**0.837**	–	–	–	–	–	–	–
4 D	–	0.104	–	**0.743**	–	0.606	**0.773**	–	–	–	–	–
5 ND	0.011	–	**0.867**	–	**0.968**	–	**0.803**	–	–	–	–	–
5 D	–	0.112	–	**0.949**	–	0.661	–	**0.808**	**0.851**	–	–	–
6 ND	0.174	–	**0.864**	–	**0.922**	–	**0.835**	–	**0.867**	–	–	–
6 D	–	**0.525**	–	**0.839**	–	**0.856**	–	**0.728**	–	**0.775**	**0.830**	–

D: dominant upper limb; ND: non-dominant upper limb. Values in bold: P<0.05 (Spearman).

### Correlation between muscle strength (MRC), JTT and MFM scores

Vignos score strongly correlated to proximal (r=-0.847; P<0.001) and distal muscle strength (r=-0.790; P<0.001). Proximal muscle strength strongly correlated to subtest 6 performed by non-dominant UL (r=-0.767; P<0.001) and to distal muscle strength (r=0.777; P<0.001). Distal muscle strength strongly correlated to subtests 3 and 6, performed by the dominant UL (r=-0.717 and r=-0.709, respectively; P<0.001). MFM D3 strongly correlated to subtest 6, performed by the dominant UL (r=-0.709; P<0.001) (Table 4).

**Table 4.** Correlations (r) between proximal and distal upper limb muscle strength, subtests of Jebsen-Taylor Test, and scores of dimension 3 of Motor Function Measure (MFM D3).

	Vignos score	Age	Proximal muscle strength	Distal muscle strength	MFM D3
Subtest 1 ND	0.099	-0.248	-0.043	-0.117	-0.117
Subtest 1 D	0.176	-0.221	-0.054	-0.147	-0.147
Dominance index 1	0.193	-0.272	-0.033	0.066	0.066
Subtest 2 ND	**0.483**	0.154	**-0.621**	**-0.621**	**-0.578**
Subtest 2 D	0.368	0.188	**-0.555**	**-0.571**	**-0.529**
Dominance index 2	0.168	0.070	-0.181	0.013	0.013
Subtest 3 ND	0.302	0.197	**-0.602**	**-0.531**	**-0.461**
Subtest 3 D	**0.420**	0.060	**-0.585**	**-0.715**	**-0.498**
Dominance index 3	0.195	-0.118	0.103	-0.099	-0.099
Subtest 4 ND	0.354	0.074	**-0.575**	**-0.494**	**-0.483**
Subtest 4 D	0.376	0.037	**-0.570**	**-0.579**	-0.366
Dominance index 4	0.084	0.168	-0.092	-0.113	-0.097
Subtest 5 ND	0.321	0.225	**-0.523**	**-0.457**	-0.330
Subtest 5 D	0.377	0.088	**-0.585**	**-0.626**	**-0.484**
Dominance index 5	0.231	-0.395	**-0.536**	**-0.464**	-0.276
Subtest 6 ND	**0.442**	0.212	**-0.767**	**-0.678**	**-0.422**
Subtest 6 D	**0.511**	0.087	**-0.649**	**-0.709**	**-0.709**
Dominance index 6	-0.192	-0.110	0.357	0.186	-0.276
Vignos score	–	0.296	**-0.847**	**-0.790**	**-0.408**
Age	0.296	–	-0.255	-0.232	0.289
Proximal muscle strength	**-0.847**	-0.255	–	**0.777**	0.304
Distal muscle strength	**-0.790**	-0.232	**0.777**	–	**0.528**
Motor function measure D3	**-0.408**	0.289	0.304	**0.528**	–

ND: non-dominant; D: dominant. Values in bold: P<0.05 (Pearson).

## Discussion

The present study investigated possible differences and relationships between the performances of dominant and non-dominant ULs on daily life, goal-directed activities of patients with DMD/BMD with normative data of healthy controls. The clinical heterogeneity of our sample was expected. We believe this phenotypic variability is important to represent the whole spectrum of UL dysfunctions observed in DMD/DMB forms and stages.

First, we hypothesized that dominant and non-dominant ULs would exhibit significantly different performances. Our results showed that this occurred only with JTT subtest 1, which was a writing task. The writing subtest was significantly faster with the dominant UL in patients and controls. This ability involves much more unilateral coordination than the other tasks (22). This difference between UL performances was not observed in the other five JTT subtests (2: turning over cards; 3: picking up small common objects; 4: simulating feeding; 5: stacking checkers; 6: picking up large light objects).

UL performance of DMD patients has been studied by Janssen et al. (6). They assessed the maximal voluntary isometric contraction of biceps brachii, anterior deltoid and lateral deltoid muscles, single joint movements of elbow flexion/extension and pronation/supination, shoulder abduction/adduction, flexion/extension, internal/external rotation and the simulation of reaching forward, touching contralateral shoulder, combing hair and bringing the hand to the mouth (6). No significant differences were found between left and right UL movements considering muscle activation. However, the authors worked with open-loop situations that have much higher predictability than the present study. In our study, patients had to deal with closed-loop tasks (24). Considering subtest 4 (simulating feeding) as an example, the task requires motor reprogramming during the attempts of holding a spoon, picking up the beans, transporting and depositing them in a bowl. The result of action is continually considered for movement corrections during the task. JTT protocol involves real goals instead of only simulating the tasks, which results in higher muscular demand (25).

Second, we hypothesized that UL asymmetry would be less evident in initial cases of dystrophinopathies (younger patients) and more evident in severe cases (older patients). This hypothesis was not confirmed by our results. Although we included wide ranges for age and Vignos scores, no correlations between age, dominance index and timed performance were observed.

We also hypothesized that UL asymmetry would be higher in patients with DMD/BMD than in healthy controls, due to the compensatory movements employed to perform functional tasks. The dominance indexes showed that patients with DMD/BMD are not more asymmetrical than gender- and age-matched healthy controls. The dominance index was developed to elucidate how much slower the non-dominant limb would be than the dominant limb, and therefore, how asymmetric patients would be. The indexes close to 1 represent the absence of asymmetry in timed performance of UL of patients and healthy controls. Furthermore, the dominance index was not correlated with age or Vignos score, showing that it is independent of disease stage.

We observed that the performances of ULs were consistently correlated in patients. This relationship can be explained by the existence of motor equivalence, which is part of the central motor program theory (26). This theory states that limbs move together, as a unit, due to a unique central motor program, which controls all action parameters. Besides, one limb activity can prevail over the other. Therefore, the non-dominant can move to assist the dominant UL. Interestingly, we found the same pattern in patients with DMD/DMB.

We found many correlations between proximal and distal muscle strength and timed performance on JTT tasks. Lower proximal and distal muscle strength denotes higher times on JTT subtests. Also, JTT times of dominant and non-dominant ULs were correlated to each other and many of them were also correlated to MFM. JTT and MFM represent different ways of assessing UL function in DMD/BMD patients. Dimension 3 of MFM assesses the quality of distal UL movements, with only one timed task, and JTT assesses timed performance, but both tests require dexterity and represent motor function. Nunes et al. (2016) found a relationship between motor function and muscular strength measured by MFM and manual testing, respectively, in patients with DMD. The present study expands this knowledge, establishing the relationship between timed performance, motor function and muscular strength.

Scoliosis is usually secondary to muscle weakness and part of the natural history of DMD (27), with higher prevalence among more severe cases (28,29). The impact of scoliosis on UL function was poorly studied in the literature. Patients with good trunk stability can perform UL functions without support. Contrarily, patients with poor trunk stability need trunk support to perform UL tasks. A recent study (30) related the occurrence of scoliosis with poor UL function, due to the impairment of sitting balance. Therefore, reduced sitting balance has a negative influence on UL function (31,32). Besides, it seems that the side scoliosis is not related to UL dominance, although the non-dominant UL is more frequently observed as a support for the non-aligned trunk side. Scoliosis convexity did not coincide with UL dominance.

Cognitive impairment is common in boys with DMD/BMD (33). Therefore, feasible tests must be developed, considering this characteristic. In a previous pilot study (10), we verified that in patients aged 10 to 30 years, MMSE scores were correlated to scores of cognitive tests (verbal fluency, clock drawing, and digits - direct and inverse orders). That study showed that patients scoring 11 or higher in MMSE can perform all JTT and MFM subtests. We observed that cognitive impairment did not interfere with the comprehension of simple verbal commands. However, lower MMSE scores were obtained in individuals with lower motor performance on JTT and MFM. Patients with better cognitive function may select more efficient motor synergies to overcome muscular weakness.

The findings provide relevant information for clinical practice. First, this assessment battery did not show any symptoms of overuse of dominant UL or disuse of non-dominant UL and can be considered safe. Second, as dominant and non-dominant UL performances were correlated, the assessment protocol can be performed with only one UL, to eliminate redundancy. It may reduce assessment time and prevent fatigue. Third, this assessment protocol evaluates two different and relevant features of UL function: timed performance (JTT) and UL movements (MFM D3).

Future studies could describe the compensatory movements performed by patients with DMD/BMD, not only on limbs, but also trunk movements, which contribute to a better performance of UL functions. Trunk movements may have helped on some JTT tasks. Besides, the investigation between limbs and trunk strength, and timed performed data may add information for better understand the performance of patients with DMD/BMD. These findings will optimize clinical intervention approaches to preserve muscle activation and UL function.

In conclusion, UL performance was only asymmetrical in the writing test, but not in other UL tasks. Dominant and non-dominant UL performances were correlated on daily life, goal-directed activities of patients with DMD/BMD. Gender- and age-matched healthy controls were not more asymmetrical than patients. Asymmetry was not more evident in more severe cases (older patients) than in less severe cases (younger patients). Correlations between timed performance, motor function and muscle strength were found, but age was not correlated to these variables.

## References

[1] Janssen MM, Bergsma A, Geurts AC, de Groot IJ (2014). Patterns of decline in upper limb function of boys and men with DMD: an international survey. J Neurol.

[2] Alemdaroğlu I, Karaduman A, Yilmaz ÖT, Topaloğlu H (2015). Different types of upper extremity exercise training in Duchenne muscular dystrophy: effects on functional performance, strength, endurance, and ambulation. Muscle Nerve.

[3] Mcdonald CM, Abresch RT, Carter GT, Fowler WM, Johnson ER, Kilmer DD (1995). Profiles of neuromuscular diseases. Duchenne muscular dystrophy. Am J Phys Med Rehabil.

[4] Henricson EK, Abresch RT, Cnaan A, Hu F, Duong T, Arrieta A (2013). The cooperative international neuromuscular research group Duchenne natural history study: glucocorticoid treatment preserves clinically meaningful functional milestones and reduces rate of disease progression as measured by manual muscle testing and other commonly used clinical trial outcome measures. Muscle Nerve.

[5] Servais L, Deconinck N, Moraux A, Benali M, Canal A, Van Parys F (2013). Innovative methods to assess upper limb strength and function in non-ambulant Duchenne patients. Neuromuscul Disord.

[6] Janssen MM, Harlaar J, de Groot IJ (2015). Surface EMG to assess arm function in boys with DMD: a pilot study. J Electromyogr Kinesiol.

[7] Connolly AM, Malkus EC, Mendell JR, Flanigan KM, Miller JP, Schierbecker JR (2015). Outcome reliability in non-ambulatory boys/men with Duchenne muscular dystrophy. Muscle Nerve.

[8] Juan-Mateu J, Gallano P, Trujillo-Tiebas MJ, Grupo AEGH/CIBERER (2012). Recommendations of good practices for the genetic diagnosis of Duchenne andBecker muscular dystrophies. Med Clin.

[9] Folstein MF, Folstein SE, McHugh PR (1975). Mini-Mental State: a practical method for grading the cognitive state of patients for clinician. J Psychiatr Res.

[10] Voos M, Favero F, Dias K, Artiheiro M, Oliveira A, Caromano F (2015). Dissociation between motor and cognitive skills in patients with Duchenne muscular dystrophy. Neuromuscul Disord.

[11] Vignos PJ, Spencer GE, Archibald KC (1963). Management progessive muscular distrophy of childhood. JAMA.

[12] Brooke MH, Griggs RC, Mendell JR, Fenichel GM, Shumate JB, Pellegrino RJ (1981). Clinical trial in duchenne dystrophy: I design of protocol. Muscle Nerve.

[13] Oldfield RC (1971). The assessment and analysis of handedness: the Edinburgh inventory. Neuropsychol.

[14] Scott OM, Hyde SA, Goddard C, Dubowitz V (1982). Quantification of muscle function in children: a prospective study in Duchenne muscular dystrophy. Muscle Nerve.

[15] Nunes MF, Hukuda ME, Favero FM, Voos MC, Oliveira AB, Caromano FC (2016). Relationship between muscle strength and motor function in Duchenne muscular dystrophy. Arq Neuropsiquiatr.

[16] Hsu JD, Quinlivan R (2013). Scoliosis in Duchenne muscular dystrophy. Neuromuscul Disord.

[17] Jebsen RH, Taylor N, Triechmann RB, Trotter MJ, Howard LA (1969). An objective and standardized test of hand function. Arch Phys Med Rehabil.

[18] Ferreiro KN, Santos RL, Conforto AB (2010). Psychometric properties of the Portuguese version of the Jebsen-Taylor test for adults with mild hemiparesis. Braz J Phys Ter.

[19] Bérard C, Payan C, Hodgkinson I, Fermanian J (2005). A motor function measure scale for neuromusculular diseases. Construction and validation study. Neuromuscular Disord.

[20] Bérard C, Payan C, Fermanian J, Girardot F, Grouped’Etude MFM (2006). A motor function measurement scale for neuromuscular disease - description and validation study. Rev Neurol.

[21] Iwabe C, Miranda-Pfeilsticker BH, Nucci A (2008). Medida da função motora: versão da escala para o português e estudo de confiabilidade. Rev Bras Fisioter.

[22] Sarafraz Z, Vahedi Z (2008). Hand function related to age and gender. Iran Rehab J.

[23] McGraw KO, Wong SP (1192). A common language effect-size statistic. Psychol Bull.

[24] Adams JA (1971). A closed-loop theory of motor learning. J Motor Behav.

[25] McKay JL, Ting LH (2012). Optimization of muscle activity for task-level goals predicts complex changes in limb forces across biomechanical contexts. PLoS Comput Biol.

[26] Schmidt RA, Lee TD (2011). Motor control and learning: a behavioral emphasis.

[27] Thong M-K, RajzBazlin RI, Wong K-T (2005). Diagnosis and management of Duchenne muscular dystrophy in a developing country over a 10-year period. Dev Med Child Neurol.

[28] Boulay C, Finidori G (2015). Aspects fonctionnel set orthopédiques des dystrophinopathies. Arch Pédiatr.

[29] Scannell BP, Yaszay B, Bartley CE, Newton PO, Mubarak SJ (2016). Surgical correction of scoliosis in patients with duchenne muscular dystrophy: 30-year experience. J Pediatr Orthop.

[30] Janssen MM, Hendriks JC, Geurts AC, de Groot IJ (2016). Variables associated with upper extremity function in patients with Duchenne muscular dystrophy. J Neurol.

[31] Chen CL, Yeung KT, Bih LI, Wang CH, Chen MI, Chien JC (2013). The relationship between sitting stability and functional performance in patients with paraplegia. Arch Phys Med Rehabil.

[32] Flatters I, Mushtaq F, Hill LJ, Holt RJ, Wilkie RM, Mon-Williams M (2013). The relationship between a child’s postural stability and manual dexterity. Exp Brain Res.

[33] Connolly AM, Florence JM, Cradock MM, Malkus EC, Schierbecker JR, Siener CA (2013). Motor and cognitive assessment of infants and young boys with Duchenne Muscular Dystrophy: results from the Muscular Dystrophy Association DMD Clinical Research Network. Neuromuscul Disord.

